# BDNF in ventrolateral orbitofrontal cortex to dorsolateral striatum circuit moderates alcohol consumption, seeking and relapse

**DOI:** 10.1038/s41386-025-02274-1

**Published:** 2025-11-09

**Authors:** Sowmya Gunasekaran, Jeffrey J. Moffat, Joshua D. Epstein, Khanhky Phamluong, Yann Ehinger, Dorit Ron

**Affiliations:** Alcohol and Addiction Research Group, Department of Neurology, University of California San Francisco, San Francisco, CA USA

**Keywords:** Addiction, Neurotrophic factors

## Abstract

BDNF plays a crucial role in shaping the structure and function of neurons. In rodents, BDNF signaling in the dorsolateral striatum (DLS) is part of an endogenous pathway that protects against the development of phenotypes associated with alcohol use. Dysregulation of BDNF levels in the cortex or dysfunction of BDNF/TrkB signaling in the DLS of rodents results in the escalation of alcohol drinking and compulsive alcohol intake. The major source of BDNF in the striatum is the prefrontal cortex. We identified a small ensemble of BDNF-positive neurons in the mouse ventrolateral orbitofrontal cortex (vlOFC), a region implicated in alcohol use disorder (AUD), that extend axonal projections to the DLS, which is associated with alcohol drinking behaviors. We speculated that BDNF in vlOFC-to-DLS circuit may play a role in limiting alcohol drinking and that heavy alcohol intake disrupts this protective pathway. We found that *BDNF* expression is reduced in the vlOFC of male but not female mice after long-term cycles of binge alcohol drinking and withdrawal. We further discovered that overexpression of BDNF in vlOFC-to-DLS but not in vlOFC-to-dorsomedial striatum (DMS) or M2 motor cortex-to-DLS circuit reduces alcohol but not sucrose intake and preference. We further showed that BDNF in vlOFC-to DLS reduces alcohol self-administration, alcohol seeking, and relapse. Finally, we found that systemic administration of BDNF receptor TrkB agonist, LM22A-4, dampens habitual alcohol seeking. Together, our data suggest that BDNF in a small ensemble of vlOFC-to-DLS neurons may gate alcohol drinking behaviors by attenuating habitual alcohol seeking.

## Introduction

The brain-derived neurotrophic factor (BDNF) belongs to the neurotrophins family [1]. BDNF plays a vital role in neuronal differentiation and maturation [2], synaptic plasticity, learning, and memory [3]. BDNF is highly expressed in the adult rodent brain [4, 5]. The majority of BDNF is stored in presynaptic dense core vesicles and is released in an activity-dependent manner upon neuronal depolarization [6]. BDNF binds to tropomyosin receptor kinase B (TrkB) receptor, which activates PI3K/AKT, and/or PLC/PKC, and/or ERK1/2 signaling, leading to the activation of transcription [7] or translation [8]. Dysregulation of BDNF signaling has been implicated in psychiatric disorders, such as depression [9], schizophrenia [9], and addiction [10].

Only 10–15% of alcohol users develop AUD [11], implying that there are protective mechanisms that prevent the development of the disorder in the majority of the population. Using rodents as a model system, we and others, presented data to suggest that BDNF is part of a protective mechanism that gates the development of heavy alcohol intake and abuse (reviews [12–14]). For example, we found that the activation of BDNF/TrkB/ERK1/2 but not PI3K/AKT, or PLC/PKC signaling in the DLS, a region involved in habitual behavior [15], keeps alcohol intake in moderation by activating the transcription machinery [16–19].

The prefrontal cortex (PFC) is the major source of BDNF in the striatum [20–23], and we and others, reported that escalation of alcohol intake results from a breakdown in BDNF signaling. Specifically, chronic high alcohol consumption attenuates *BDNF* expression in corticostriatal regions of rodents [24–26], which is mediated by the microRNA machinery in the medial prefrontal cortex (mPFC) [25, 26], resulting in escalation of alcohol consumption [25, 26]. We further found that the transition from moderate to heavy alcohol intake is also mediated by the alterations in the membranal localization of the BDNF receptors, TrkB and p75NTR in the DLS of rats [27]. Finally, we reported that transgenic mice carrying a polymorphism within the BDNF gene that disrupts BDNF release [28], compulsively drink alcohol, exhibit a reduction in the anxiolytic actions of alcohol and an increase in alcohol preference over social interaction in mice [29, 30]. Together, these data suggest that BDNF in corticostriatal regions gates alcohol drinking behaviors, and that AUD-like phenotypes develop in part when BDNF signaling ceases to function.

The cortical regions that release BDNF into the DLS have not been carefully mapped out. Using a combination of transgenic mouse lines together with a viral-mediated retrograde tracing strategy, we characterized BDNF-expressing cortical neurons that project to the DLS [23]. We found that a small ensemble of BDNF-positive neurons in the vlOFC extend axonal projections to the DLS [23]. The OFC plays a critical role in decision-making, reward-prediction error [31–33], reward information [34], and stimulus-outcome behaviors [35–37]. The OFC has also been identified as a critical region in AUD [38, 39]. Specifically, in humans, alcohol dependence reduces white matter and neuronal density in the OFC [40–42], and the connectivity between OFC and striatum is altered in abstinent subjects [43]. In rodents, alcohol affects the activity of OFC neurons [44, 45]. Furthermore, OFC lesions or chemogenetic inhibition increase alcohol drinking [46–48] and decrease context and cue-induced reinstatement [49, 50]. Together, these data suggest that the OFC is an important target of alcohol. However, whether BDNF in vlOFC-to-DLS projecting neurons affects alcohol drinking behaviors is unknown. We report that *BDNF* expression is reduced in the vlOFC of mice following 7 weeks of intermittent access to 20% alcohol in a 2-bottle choice procedure (IA20%2BC). We further show that overexpression of BDNF in vlOFC-to-DLS circuit limits alcohol intake, seeking, and relapse. Finally, we show that the systemic administration of a TrkB agonist reverts habitual to goal-directed alcohol seeking.

## Materials and methods

Reagents, preparation of solutions, collection of brain samples, real-time PCR, purchasing of viruses, stereotaxic viral infection, confirmation of viral expression, and behavioral procedures can be found in the supplementary material.

### Animals

Male (152) and female (38) C57BL/6J mice (6–8 weeks) were purchased from Jackson Laboratory and were allowed one week of habituation before experiments began. Mice were individually housed on paper-chip bedding, under a reverse 12-h light-dark cycle. Temperature and humidity were kept constant at 22 ± 2°C, and relative humidity was maintained at 50 ± 5%. Mice were allowed access to food and tap water *ad libitum*. All animal procedures were approved by the University’s Institutional Animal Care and Use Committee (IACUC) and were conducted in agreement with the Association for Assessment and Accreditation of Laboratory Animal Care.

### Behavioral procedures

#### Intermittent access to 20% alcohol two-bottle choice (IA20%2BC)

IA20%2BC was conducted as previously described [51]. Briefly, mice were given one bottle of 20% alcohol (v/v) in tap water and one bottle of water for 24 h on Monday, Wednesday, and Friday, with 24 or 48-h (weekend) of alcohol withdrawal periods during which mice consumed only water. The placement of water or alcohol bottles was alternated between each session to avoid side preference. Alcohol and water bottles were weighed at the beginning and end of each alcohol drinking session, and alcohol intake (g/kg of body weight), water intake (ml/kg) and total fluid intake (ml/kg) were calculated. Two bottles containing water and alcohol in an empty cage were used to evaluate the spillage. Alcohol preference ratio was calculated by dividing the volume of alcohol consumed to the total volume of fluid intake.

#### Operant alcohol self-administration

Alcohol operant self-administration training and habitual alcohol seeking training was performed as described previously [52]. First, mice underwent 7 weeks IA20%2BC. Mice drinking more than 12.5 g/kg were selected for the experiment. Alcohol operant self-administration training was initiated under a fixed-ratio (FR) 1 schedule, i.e., one lever press resulted in the delivery of one reward, for four 6-h FR1 sessions, followed by four 4-h FR1 sessions, and finally four 2-h FR1 sessions. Reward deliveries were paired with a 3-s tone and illumination of a cue light. All remaining sessions lasted 2 h. Mice were then trained on a random-interval (RI) schedule, during which rewards were delivered with random delays following active lever presses according to previous studies [53–55]. Timepoints were pseudo-randomly assigned by the computer program. Mice first underwent 5 sessions on an RI30 schedule (delays averaging 30 s after lever press, with intervals ranging from 0 to 60 s). Mice were then subjected to 5 sessions of RI60 training (intervals ranging from 30 to 90 s). Mice were divided into 2 groups with similar numbers of active lever presses (106.41 ± 40.78 and 116.35 ± 58.35), port entries (96.35 ± 45.73 and 72.61 ± 14.36), and amount of self-administered alcohol (2.55 ± 0.70 and 2.46 ± 0.79 g/kg/2 h). Three mice were excluded from the operant self-administration procedure due to low pressing numbers. BDNF or mCherry control was then overexpressed in the vlOFC-to-DLS circuit. Three weeks following surgery, RI30 was resumed for 5 sessions, followed by RI60 until the end of the experiment. The number of active lever presses and reward port entries, as well as the number of reward deliveries, were recorded during each session.

LM22A-4 administration: Thirty minutes prior to a degradation session, mice received intraperitoneal (i.p.) administration of saline or LM22A-4 (100 mg/kg) [29].

#### Contingency degradation

Contingency degradation was used as previously described [56, 57] to test the sensitivity of mice to changes in the response-outcome association. The procedure was conducted across nondegraded (ND) and degraded (D) sessions. During the 2-h degraded session, a lever was extended, but lever presses produced no consequences. In total, 2-3 degradation sessions were performed. During the nondegraded sessions, alcohol deliveries occurred at a rate that was determined based on each animal’s average reward rate during the four RI60 schedules of nondegraded sessions, prior to degradation.

#### Extinction and reacquisition

Extinction and reacquisition procedures were conducted following completion of the RI training phase and contingency degradation test. Mice underwent 13 daily extinction sessions (2-h each), during which both levers were available, but lever presses were not paired with alcohol deliveries and no cues associated with reward delivery were presented.

The first extinction session was used to assess alcohol-seeking behavior in the absence of reinforcement, as measured by the number of active lever presses and port entries. Subsequent extinction sessions continued until responding stabilized across sessions and decreased to at least half of lever presses compared to previous RI sessions.

Following the last extinction session, reacquisition training (2-h) was conducted during which lever presses were paired with cues and alcohol deliveries to evaluate the reinstatement of operant responding. The session parameters were identical to FR1 schedule.

### Statistical analysis

D’Agostino–Pearson normality test, Shapiro–Wilk normality test and F-test/Levene tests were used to verify the normal distribution of variables and the homogeneity of variance, respectively. Data were analyzed using the appropriate statistical test, including two-tailed unpaired *t*-test, one-way ANOVA, two-way ANOVA with and without repeated measures followed by post-hoc test. GraphPad Prism 9 was used for statistical analyses. All data are expressed as mean +/− SEM. Significance was set at *p* < 0.05.

## Results

### High alcohol drinking reduces *BDNF* expression in the vlOFC

**Fig. 1:**
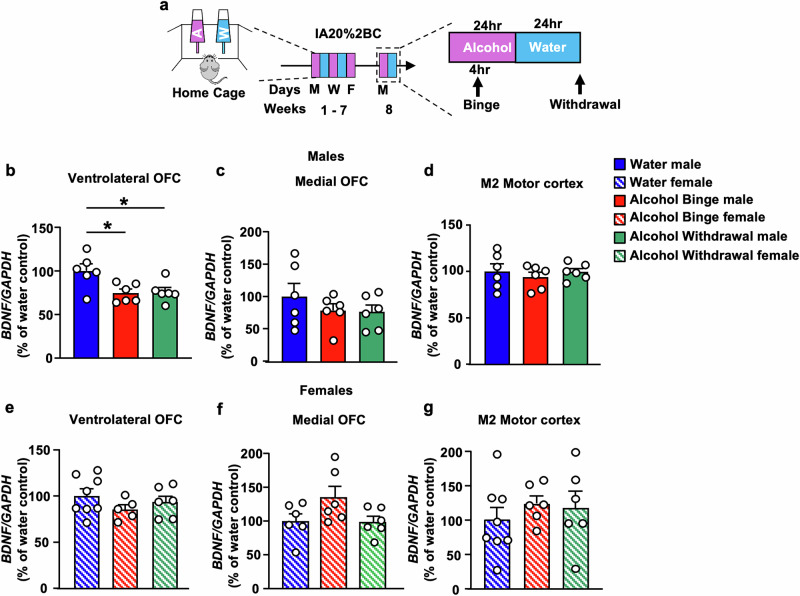
*BDNF* expression is attenuated in the vlOFC but not in the mOFC and M2 of male but not female mice in response to chronic high alcohol intake and withdrawal. **a** Timeline of experiments: Female and male mice underwent 7 weeks of IA20%2BC or water only (Supplementary Table 1). Four hours after the beginning of the last drinking session (“binge”) and 24 h after the last drinking session (“withdrawal”), the vlOFC, mOFC and motor cortex M2 were dissected and harvested. The expression of *BDNF* was measured by RT-qPCR using *GAPDH* an internal control. Data are presented as the average ratio of *BDNF* to *GAPDH* ± SEM and are expressed as percentage of water control. *BDNF* mRNA in the vlOFC (**b**), mOFC (**c**) and M2 motor cortex (**d**) of male mice. *BDNF* mRNA in the vlOFC (**e**), mOFC (**f**) and M2 motor cortex (**g**) of female mice. *p  <  0.05; ns non-significant. *n* = 6–8 per group.

We identified a small ensemble of vlOFC neurons expressing BDNF that project to the DLS of mice [23]. Since BDNF signaling in the DLS is a locus for keeping alcohol drinking in moderation [18, 19], we hypothesized that normal levels of BDNF in the OFC are required to control the development of heavy alcohol intake. We further hypothesized that breakdown in BDNF signaling in OFC-to-DLS circuit promotes the escalation of alcohol intake. To address these hypotheses, we first tested whether high alcohol drinking alters *BDNF* levels in the vlOFC. Mice underwent 7 weeks of IA20%-2BC procedure [52] (Fig. 1a). Male mice consumed an average of 14.47 ± 1 g/kg/24 h (Supplementary Fig. 1a–c; Supplementary Table 1), and female mice drank an average of 19.27 ± 1.1 g/kg/24 h (Supplementary Fig. 1d–f; Supplementary Table 1). Mice were sacrificed 4 h after the beginning of the last drinking session (“binge”) and 24 h after the last drinking session (“withdrawal”), and *BDNF* expression was measured. We found that *BDNF* mRNA in the vlOFC was significantly decreased in male mice that were subjected to 7 weeks of IA20%2BC during both binge and withdrawal as compared to mice consuming water only (Fig. 1b) (One-way ANOVA: effect of treatment, F_(2,15)_ = 5.705, *p* = 0.014; Post hoc Dunnett’s multiple comparison test indicates significant differences between water and binge drinking and between water and withdrawal). We further discovered that alcohol-mediated reduction in *BDNF* mRNA levels is localized to the vlOFC since no changes in *BDNF* expression were detected in the medial OFC (mOFC) (Fig. 1c) (One-way ANOVA: effect of treatment, F_(2,15)_ = 0.846, *p* = 0.448). As BDNF neurons in M2 motor cortex send dense projections to the DLS [23], we examined the level of *BDNF* mRNA in this region in response to high alcohol consumption. As shown in Fig. 1d, *BDNF* mRNA levels in M2 motor cortex of male mice were unchanged by alcohol (One-way ANOVA: effect of treatment, F_(2,15)_ = 0.354, *p* = 0.707). Interestingly, the alterations of *BDNF* levels were sex specific as *BDNF* mRNA levels in the vlOFC, mOFC or M2 of female mice were unaltered after binge drinking and withdrawal from 7 weeks of IA20%2BC (Fig. 1e–g) (one-way ANOVA: vlOFC, effect of treatment, F_(2,16)_ = 1.13, *p* = 0.347; mOFC, effect of treatment, F_(2,15)_ = 2.88, *p* = 0.087; M2 motor cortex, effect of treatment, F_(2,17)_ = 0.484, *p* = 0.624). Together, these data suggest that chronic alcohol consumption reduces *BDNF* expression specifically in the vlOFC of male mice. As a result, all subsequent experiments were performed in male mice.

### DIO-Cre-dependent overexpression of BDNF in vlOFC neurons projecting to the DLS

**Fig. 2:**
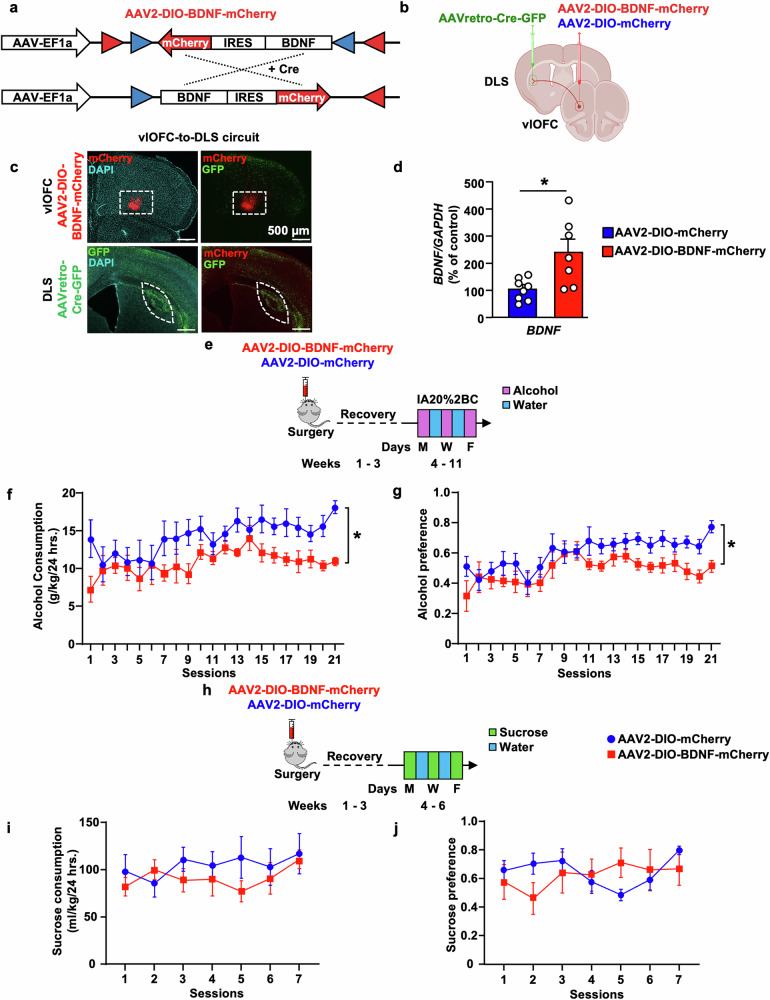
Overexpression of BDNF in vlOFC-to-DLS circuit moderates alcohol but not sucrose intake. **a** Viral strategy of Cre-dependent overexpression of BDNF: BDNF and mCherry coding sequences are floxed by a pair of loxP (blue triangles) and lox2272 (red triangles) sites. In the absence of Cre recombinase, the BDNF and mCherry coding sequences are inverted relative to the EF1a promoter. When expressed, Cre recombinase inverts the BDNF and mCherry sequences into a correct orientation, allowing their expression. **b** Schematic representation of BDNF overexpression in vlOFC-to-DLS circuit. Mice received bilateral injections of AAV2-DIO-BDNF-mCherry or AAV2-DIO-mCherry in the vlOFC and AAVretro-Cre-GFP in the DLS. **c** Representative images depicting targeting of AAV2-DIO-BDNF-mCherry (red) in the vlOFC and AAVretro-Cre-GFP (green) in the DLS. Red signal indicates expression of Cre and infection of AAV2-DIO-BDNF-mCherry, confirming the overexpression of BDNF in vlOFC neurons projecting to the DLS. Top left panel depicts mCherry (red) and DAPI (cyan). Bottom left panel depicts GFP (green) and DAPI (cyan). Top and bottom right panels depict mCherry (red) and GFP (green). **d** The vlOFC was dissected 10 weeks after the infusion of AAV2-DIO-BDNF-mCherry or AAV2-DIO-mCherry in the vlOFC and retroAAV-Cre-GFP in DLS and *BDNF* expression was measured by RT-qPCR. *GAPDH* was used as an internal control. Data are presented as the average ratio of *BDNF* to *GAPDH* ± SEM and expressed as the percentage of water control. **e** Timeline of experiments: Three weeks after the surgery, mice underwent IA20%2BC for 7 weeks in the home cage (Supplementary Table 1). **f** Alcohol intake was recorded. **g** Alcohol preference was calculated as the ratio of alcohol intake relative to total fluid intake. **h** Timeline of experiments: Mice received a bilateral injection of AAV2-DIO-BDNF-mCherry or AAV2-DIO-mCherry in the vlOFC and AAVretro-Cre-GFP in the DLS and three weeks after the viral injection, mice were subjected to 2-bottle choice with 0.3% sucrose drinking for 7 sessions in the home cage (Supplementary Table 1). **i** Sucrose intake was recorded. **j** Sucrose preference was calculated as the ratio of sucrose intake relative to total fluid intake. Data are represented as mean ± SEM. *p < 0.05, ns: non-significant. *n* = 6–8 per group.

As described above, high alcohol drinking downregulates *BDNF* expression in the vlOFC. If BDNF in vlOFC-to-DLS circuit is gating alcohol intake, then replenishing its levels in this circuitry will revert high alcohol intake to moderate levels. To test this possibility, we utilized a circuit-specific strategy to overexpress BDNF in vlOFC neurons that project to the DLS by using the DIO/Cre system, enabling *BDNF* expression only in the presence of Cre recombinase (Fig. 2a) [23]. AAV2-DIO-BDNF-mCherry virus (1 ×10^12^ gc/ml) was bilaterally infused into the vlOFC and AAVretro-Cre-GFP virus (3 ×10^12^ vg/ml) was bilaterally infused into the DLS (Fig. 2b, c). This allowed Cre expression in vlOFC neurons projecting to the DLS visualized by GFP  (Fig. 2c), thereby activating Cre-mediated expression of BDNF visualized by mCherry in the vlOFC (Fig. 2c). We further analyzed BDNF signal in vlOFC neurons that project to the DLS in the presence of Cre recombinase (Supplementary Fig. 2a–d). In addition, we assessed the spread of both viruses in the DLS and OFC. As shown in Supplementary Fig. 3, the AAVretro-Cre-GFP virus is localized 1 mm from bregma on the AP axis in the DLS. The AAV2-DIO-BDNF-mCherry virus infection site is localized at 2.1 mm from bregma on the AP axis in the vlOFC, confirming that BDNF is overexpressed in the vlOFC to DLS circuit. Control Mice were bilaterally infected with AAV2-DIO-mCherry in the vlOFC and AAVretro-Cre-GFP in the DLS. Finally, we tested whether *BDNF* mRNA levels are elevated over baseline in vlOFC neurons that project to the DLS. As shown in Fig. 2d, there was a significant increase of *BDNF* mRNA levels in the vlOFC of mice infected with AAV2-DIO-BDNF-mCherry as compared to mice infected with AAV2-DIO-mCherry (Unpaired t-test: t (13) = 2.990, *p* = 0.010).

### BDNF in vlOFC-to-DLS projecting neurons gates alcohol but not sucrose intake

Three weeks following viral infection enabling maximal BDNF expression, mice were subjected to 7 weeks of IA20%2BC or water only (Fig. 2e). We found that overexpression of BDNF in vlOFC to DLS projecting neurons significantly reduces alcohol drinking and preference as compared to control mice (Fig. 2f, g, Supplementary Table 1) (Alcohol intake: Two-Way mixed-effect ANOVA, effect of BDNF overexpression, F_(1, 13)_ = 5.89, *p* = 0.030, effect of session, F_(5.873, 73.71)_ = 3.016, *p* = 0.011, main effect of interaction, F_(20,251)_ = 0.925, *p* = 0.554. Alcohol preference: effect of BDNF overexpression, F_(1, 13)_ = 5.91, *p* = 0.020, effect of session, F_(20,240)_ = 4.35, ****p < 0.0001, effect of interaction, F_(20, 240)_ = 0.96638, *p* = 0.507). Furthermore, alcohol intake of control mice escalated over time, whereas progressive increase of intake was not detected in mice infected with BDNF in vlOFC neurons projecting to the DLS (Fig. 2f, g). Overexpression of BDNF in vlOFC to DLS projecting neurons did not affect water and total fluid intake (Supplementary Fig. 4a, b) (Water consumption: Two-Way mixed-effect ANOVA, effect of BDNF overexpression, F_(1, 13)_ = 2.906, *p* = 0.11, effect of session, F_(20, 245)_ = 4.437, ****p < 0.0001, effect of interaction F_(20,245)_ = 0.92, *p* = 0.55; Total intake consumption: effect of BDNF overexpression, F_(1, 13)_ = 1.168, *p* = 0.2994, effect of session, F_(20, 245)_ = 1.875, *p = 0.0148, effect of interaction F_(20,245)_ = 1.536, *p* = 0.0701). Together, these data indicate that escalation of alcohol intake is due in part to the attenuation of BDNF levels in the vlOFC, which is rescued by replenishing BDNF in vlOFC-to-DLS projecting neurons. Our data further suggests that BDNF in OFC to DLS circuitry gates escalation of high alcohol intake.

Next, we set out to determine whether BDNF in vlOFC-to-DLS circuit gates the consumption of sucrose, a natural rewarding substance. To do so, a new cohort of mice was subjected to bilateral injections of AAV2-DIO-BDNF-mCherry in the vlOFC and AAVretro-Cre-GFP in the DLS. Control mice were infected with AAV2-DIO-mCherry in the vlOFC and AAVretro-Cre-GFP in the DLS. Three weeks post-viral infusion, mice underwent intermittent access to 0.3% sucrose 2BC procedure for 2 weeks (Fig. 2h). We found that *BDNF* overexpression in vlOFC-to-DLS circuit does not alter sucrose intake and preference compared to control mice (Fig. 2i, j, Supplementary Table 1) (Sucrose intake: Two-Way ANOVA, effect of BDNF overexpression, F_(1, 11)_ = 0.233, *p* = 0.638, effect of session, F_(6, 63)_ = 1.426, *p* = 0.218, effect of interaction, F_(6,63)_ = 1.605, *p* = 0.160; Sucrose preference: effect of BDNF overexpression, F_(1, 11)_ = 0.011, *p* = 0.915, effect of session, F_(6, 58)_ = 1.333, *p* = 0.257, effect of interaction, F_(6, 58)_ = 3.069, *p* = 0.012). BDNF overexpression in vlOFC-to-DLS circuit did not alter water consumption and total fluid consumption (Supplementary Fig. 4c, d) (Water intake: Two-Way mixed-effect ANOVA, effect of BDNF overexpression, F_(1, 11)_ = 0.2527, *p* = 0.62, effect of session, F_(6, 58)_ = 0.8752, *p* = 0.51, effect of interaction, F_(6, 58)_ = 2.479, *p = 0.03; Total fluid consumption: effect of BDNF overexpression, F_(1, 11)_ = 2.491, *p* = 0.1428, effect of session, F_(6, 58)_ = 1.541, *p* = 0.1811, effect of interaction, F_(6, 58)_ = 0.4101, *p* = 0.8694). Thus, the attenuation of alcohol drinking by BDNF in the vlOFC-to-DLS circuit is not due to changes in palatability and is specific to alcohol. As the striatum plays a role in motor skills [58], we determined whether overexpression of BDNF in vlOFC-to-DLS projecting neurons is due to attenuation of locomotion. As shown in Supplementary Fig. 5, total distance traveled (Supplementary Fig. 5b, c) and velocity **(**Supplementary Fig. 5d) were similar in the two groups (Total distance traveled: Mann–Whitney Test: U = 15, *p* = 0.445; Average velocity: Mann–Whitney Test: U = 15, *p* = 0.445). These results indicate that the behavioral difference in alcohol consumption is not due to changes in motor behavior.

### BDNF in vlOFC-to-DMS or M2-to-DLS projecting neurons does not moderate alcohol intake

**Fig. 3:**
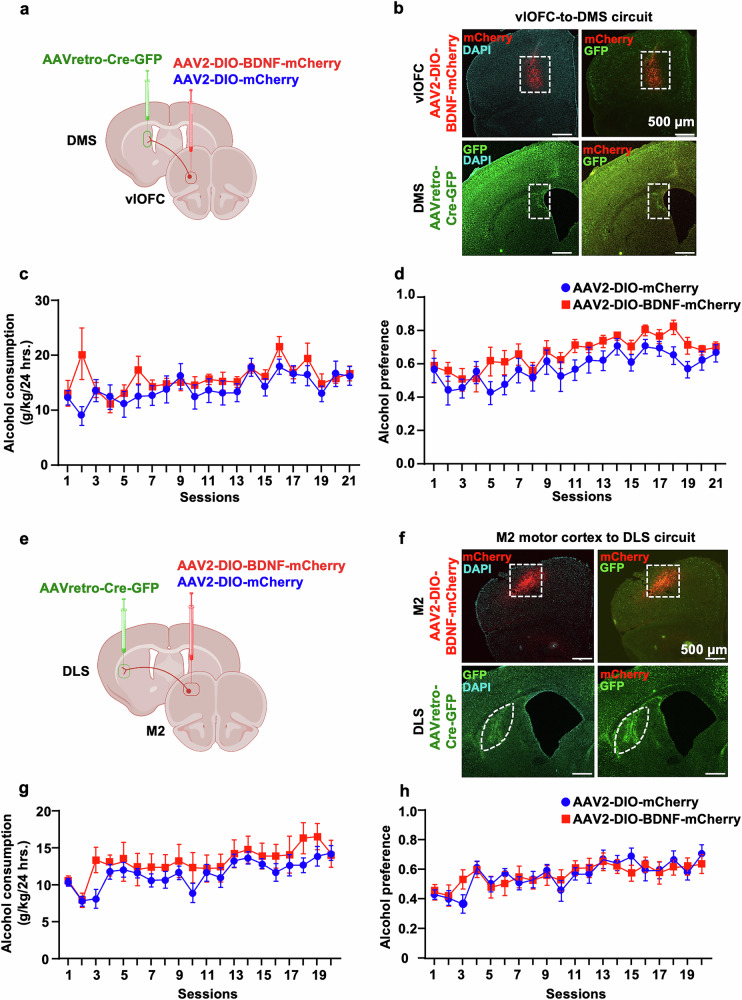
Overexpression of BDNF in vlOFC-to-DMS or M2-to-DLS neurons does not alter alcohol intake. **a**–**d** Overexpression of BDNF in vlOFC-to-DMS projecting neurons. **a** Schematic representation of BDNF overexpression in vlOFC-to-DMS circuit. Mice received bilateral injections of AAV2-DIO-BDNF-mCherry or AAV2-DIO-mCherry in the vlOFC and AAVretro-Cre-GFP in the DMS. **b** Representative image depicting targeting of AAV2-DIO-BDNF-mCherry in vlOFC and AAVretro-Cre-GFP in the DMS. Red cells indicate the expression of Cre and infection of AAV2-DIO-BDNF-mCherry, confirming the overexpression of BDNF in vlOFC neurons projecting to the DMS. Top left panel depicts mCherry (red) and DAPI (cyan). Bottom left panel depicts GFP (green) and DAPI (cyan). Top and bottom right panels depict mCherry (red) and GFP (green). **c** Three weeks after the surgery, mice underwent IA20%2BC for 7 weeks in the home cage (Supplementary Table 1), and alcohol intake was recorded. **d** Alcohol preference was calculated as the ratio of alcohol intake relative to total fluid intake. **e**–**h** Overexpression of BDNF in M2-to-DLS projecting neurons. **e** Schematic representation of BDNF overexpression in M2-to-DLS circuit. Mice received bilateral injections of AAV2-DIO-BDNF-mCherry or AAV2-DIO-mCherry in M2 and AAVretro-Cre-GFP in the DLS. **f** Representative image depicting targeting of AAV2-DIO-BDNF-mCherry in M2 and AAVretro-Cre-GFP in the DLS. Red cells indicate expression of the Cre and infection with AAV2-DIO-BDNF-mCherry confirming the overexpression of BDNF in M2 neurons projecting to the DLS. Top left panel depicts mCherry (red) and DAPI (cyan). Bottom left panel depicts GFP (green) and DAPI (cyan). Top and bottom right panels depict mCherry (red) and GFP (green). **g**) Three weeks after the surgery, mice underwent IA20%2BC for 7 weeks in the home cage (Supplementary Table 1), and alcohol intake was recorded. **h** Alcohol preference was calculated as the ratio of alcohol intake relative to total fluid. Data are represented as mean ± SEM. *n* = 8–10 per group.

A small population of BDNF projecting neurons from the vlOFC also projects to the DMS [23]. Therefore, we investigated whether BDNF in vlOFC-to-DMS projecting neurons modulates alcohol consumption. We used the same circuit-specific viral approach, consisting of a bilateral infusion of AAV2-DIO-BDNF-mCherry in the vlOFC and AAVretro-Cre-GFP in the DMS (Fig. 3a), enabling overexpression of BDNF specifically in vlOFC-DMS projecting neurons (Fig. 3b). Control mice were infected with AAV2-DIO-mCherry in the vlOFC and AAVretro-Cre-GFP in the DMS. Three weeks post-viral administration, mice underwent IA20%2BC for 7 weeks, and alcohol intake was assessed. We found no significant difference in alcohol (Fig. 3c, Supplementary Table 1), water, total fluid intake (Supplementary Fig. 6a, b), and alcohol preference (Fig. 3d) between BDNF-overexpressing mice and control groups (Alcohol intake: Two-Way mixed-effect ANOVA, effect of BDNF overexpression, F_(1, 17)_ = 1.033, *p* = 0.323, effect of session, F_(20, 330)_ = 3.750, ****p < 0.0001, effect of interaction, F_(20,330)_ = 1.592, *p* = 0.052; Alcohol preference: effect of BDNF overexpression, F_(1, 17)_ = 2.577, *p* = 0.1268, effect of session, F_(20, 327)_ = 6.605, ****p < 0.0001, effect of interaction, F_(20,327)_ = 0.5599, *p* = 0.9377; Water consumption: effect of BDNF overexpression, F_(1, 17)_ = 2.14, *p* = 0.16, effect of session, F_(20, 329)_ = 7.296, ****p < 0.0001, effect of interaction, F_(20, 329)_ = 0.38, *p* = 0.99; Total fluid consumption: effect of BDNF overexpression, F_(1, 17)_ = 0.2219, *p* = 0.6436, effect of session, F_(20, 324)_ = 4.744, ****p < 0.0001, effect of interaction, F_(20, 324)_ = 1.523, *p* = 0.0713). Together, our results suggest that BDNF in vlOFC-to-DLS but not vlOFC-to-DMS circuit moderates alcohol drinking.

BDNF-expressing neurons in M2, a region essential for motor learning and behaviors [59], extend dense projections to the DLS [23]. We, therefore, assessed whether BDNF in M2-to-DLS projecting neurons alters alcohol consumption. To do so, AAV2-DIO-BDNF-mCherry was infused into the M2 and AAVretro-Cre-GFP into the DLS (Fig. 3e, f). Control mice were infected with AAV2-DIO-mCherry in the M2 and AAVretro-Cre-GFP in the DLS. We found that BDNF overexpression in M2 neurons that project to the DLS does not alter alcohol intake (Fig. 3g, Supplementary Table 1) and preference (Fig. 3h) (Alcohol intake: Two-Way mixed-effect ANOVA, effect of BDNF overexpression, F_(1, 16)_ = 0.8408, *p* = 0.372, effect of session, F_(19, 294)_ = 5.445, ****p < 0.0001, effect of interaction, F_(19,294)_ = 0.709, *p* = 0.809; Alcohol preference: effect of BDNF overexpression, F_(1, 16)_ = 0.0001175, *p* = 0.991, effect of session, F_(19, 299)_ = 5.541, ****p < 0.0001, effect of interaction, F_(19,299)_ = 0.918, *p* = 0.560). Water and total fluid consumption were unchanged between the groups (Supplementary Fig. 6c, d) (Water consumption: Two-Way mixed-effect ANOVA, effect of BDNF overexpression, F_(1, 16)_ = 0.5373, *p* = 0.47, effect of session, F_(19, 298)_ = 6.149, ****p < 0.0001, effect of interaction, F_(19, 298)_ = 0.6853, *p* = 0.83; Total fluid consumption: effect of BDNF overexpression, F_(1, 16)_ = 3.591, *p* = 0.0763, effect of session, F_(19, 299)_ = 4.755, ****p < 0.0001, effect of interaction, F_(19, 299)_ = 0.5461, *p* = 0.9399). These data indicate that, unlike BDNF in vlOFC-to-DLS circuitry, BDNF in neurons that project from M2-to-DLS do not contribute to mechanisms regulating alcohol drinking.

### BDNF in vlOFC-to-DLS projecting neurons gates alcohol self-administration, alcohol seeking and relapse

**Fig. 4:**
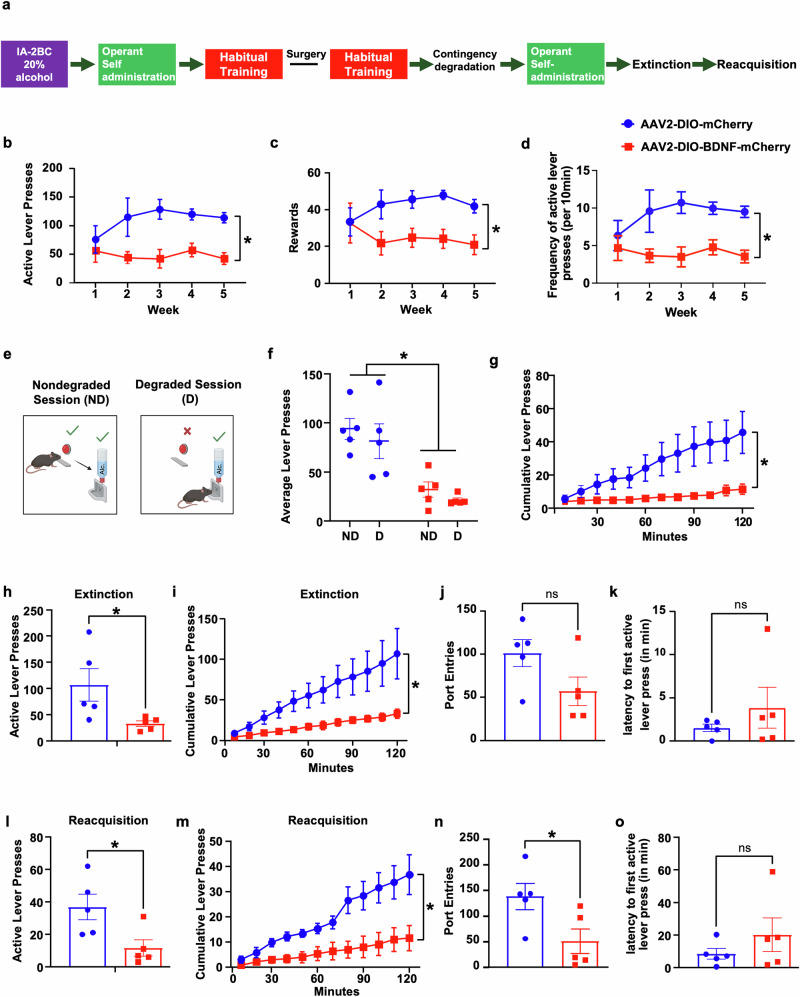
BDNF in vlOFC-to-DLS circuit moderates habitual alcohol seeking. **a** Experimental timeline: mice underwent 7 weeks of IA20%2BC alcohol in their home cage (Supplementary Table 1). Mice were then trained to operant self-administer 20% alcohol for four weeks on an FR1 schedule followed by an initial habitual alcohol seeking training using random interval (RI) training schedule. Mice were pseudo-randomly assigned to two groups. Control group received bilateral infusions of AAV2-DIO-mCherry in the vlOFC and AAVretro-Cre-GFP in the DLS. The second group received AAV2-DIO-BDNF-mCherry in the vlOFC and AAVretro-Cre-GFP in the DLS. Three weeks after surgery, RI training was resumed followed by contingency degradation testing. Mice then underwent extinction. Lever presses were recorded during the first extinction session and during a reinstatement session. **b**–**d** Operant self-administration. Average of active lever presses (**b**), rewards (**c**) frequency of active lever presses (**d**) during the operant training sessions 3 weeks after surgery. **e** Contingency degradation test: non degraded (ND) and degraded (D). During ND sessions, rewards were delivered on an RI schedule. During D sessions, alcohol rewards were delivered at a rate equal to the average of the last week of training. **f**, **g** Average of active lever presses (**f**) during ND and D testing sessions in RI schedule of reinforcement of AAV-DIO-mCherry infected and AAV-DIO-BDNF-mCherry infected mice. **g** Cumutative active lever presses of AAV-DIO-mCherry infected and AAV-DIO-BDNF-mCherry infected mice during the 2 hr degradation session. Extinction: the total number of lever presses (**h**), cumulative number of lever presses (**i**), port entries (**j**), and the latency to the first active lever press (**k**) were recorded. Reacquisition: the total number of lever presses (**l**), cumulative number of lever presses (**m**), port entries (**n**), and the latency to the first active lever press (**o**) were recorded. Data represented as mean ± SEM. *p < 0.05. *n* = 5 per group.

The DLS as well as the OFC have been implicated in habit formation and habitual drug seeking [55, 57, 60–63]. We therefore investigated the role of BDNF in the vlOFC-to-DLS circuit in habitual alcohol seeking. We utilized a contingency degradation procedure to examine habitual alcohol seeking [52]. Mice first underwent IA20%2BC for 7 weeks (Supplementary Fig. 7a–d, Supplementary Table 1) and were then trained to operantly self-administer alcohol by lever pressing for 20% alcohol using a random interval (RI) training of reinforcement (Timeline, Fig. 4a), which biases lever responding toward habitual actions [53–55]. Alcohol lever presses were similar in mice that were pre-assigned to be infected with AAV2-DIO-BDNF-mCherry or AAV2-DIO-mCherry in the vlOFC and AAVretro-Cre-GFP in the DLS (Supplementary Fig. 7e) (Two-way RM ANOVA: effect of virus F_(1,8)_ = 0.1851, *p* = 0.6783; effect of session F_(2.538,12.3)_ = 0.7956, *p* = 0.4422; effect of interaction F_3,24)_ = 0.2797, *p* = 0.8395). Following the initial training, mice were bilaterally infused with AAV2-DIO-BDNF-mCherry or AAV2-DIO-mCherry in the vlOFC and AAVretro-Cre-GFP in the DLS (Fig. 4a). Three weeks after the surgery, mice underwent 5 additional sessions of RI30 training followed by 5 sessions of RI60 training. First, we examined whether overexpression of BDNF in vlOFC to DLS neurons alters operant self-administration as compared to control mice. Similar to vlOFC BDNF overexpressing mice consuming alcohol in their home cage (Fig. 2) BDNF overexpression in vlOFC-to-DLS circuit robustly decreased alcohol self-administration shown by a reduction in active lever presses (Fig. 4b), alcohol rewards (Fig. 4c) and frequency of active lever presses (Fig. 4d) (Average active lever presses: Two-way RM ANOVA: effect of virus F_(1,8)_ = 8.696, *p* = 0.0185; effect of session F_(2.067,16.53)_ = 1.234, *p* = 0.3178; effect of interaction F_(4,32)_ = 2.647, *p* = 0.0514; Rewards: effect of virus F_(1,8)_ = 5.676, *p* = 0.0444; effect of session F_(2.595,20.76)_ = 0.4482, *p* = 0.6943; effect of interaction F_(4,32)_ = 2.684, *p* = 0.049; Frequency: effect of virus F_(1,8)_ = 8.696, *p* = 0.0185; effect of session F_(2.067,16.53)_ = 1.234, *p* = 0.3178; effect of interaction F_(4,32)_ = 2.647, *p* = 0.0514). These data suggest that BDNF in vlOFC neurons projecting to the DLS moderates alcohol self-administration.

With the caveat that BDNF overexpression in vlOFC to DLS circuit robustly decreases alcohol lever presses, we attempted to determine if the reduction in alcohol self-administration was due to an alteration in habitual alcohol seeking. Mice trained to self-administer alcohol in a goal-directed manner are sensitive to contingency degradation, while habitually trained mice are not [53–55]. We conducted a contingency degradation procedure and tested whether habitually trained mice continue to press a lever previously paired with alcohol reward delivery (Fig. 4e). During degradation sessions, mice received alcohol at a rate equal to their average reward rate during the 4 nondegraded self-administration sessions immediately preceding the degradation session. As shown in Fig. 4f, the control AAV2-DIO-mCherry infected mice pressed similarly during nondegraded and degraded sessions, indicating habitual responding (Two-way RM ANOVA: effect of virus F_(1,8)_ = 20.90, *p* = 0.0018; effect of session F_(1,8)_ = 2.084, *p* = 0.1868; effect of interaction F_(1,8)_ = 0.01386, *p* = 0.9092). Unfortunately, however, we were unable to assess habitual and goal-directed behavior using a contingency degradation test, as AAV2-DIO-BDNF-mCherry infected mice reduced active lever pressing during nondegraded and degraded sessions compared to control mice (Fig. 4f, g) (cumulative presses during degradation: Two-way RM ANOVA: effect of virus F_(1,8)_ = 5.639, *p* = 0.0449; effect of session F_(1.356,10.85)_ = 10.90, *p* = 0.0047; effect of interaction F_(11,88)_ = 5.542, *p* < 0.0001).

As alcohol seeking precedes alcohol drinking, we examined whether BDNF expressed in this circuitry controls alcohol seeking. To do so, we examined lever presses that were previously associated with alcohol rewards during an extinction session (Fig. 4a). As shown in Fig. 4h, i, overexpression of BDNF in vlOFC to DLS circuit significantly decreases the total number of lever presses as well as the cumulative lever presses (Average active lever presses: Mann–Whitney Test: U = 1, *p* = 0.0159; Cumulative presses: Two-way RM ANOVA: effect of virus F_(1,8)_ = 5.897, *p* = 0.0413; effect of session F_(1.081,8.645)_ = 17.13, *p* = 0.0025; effect of interaction F_(11,88)_ = 5.023, *p* < 0.0001).

Relapse to alcohol and drug seeking can be modeled by a reacquisition test in which animals first go through a period of extinction in which alcohol deliveries are no longer paired with active lever presses. During the reacquisition test, mice reacquire operant responding [64]. We determined whether overexpressing BDNF in the vlOFC to DLS circuitry alters alcohol lever presses during a reacquisition session. To do so, mice self-administering alcohol underwent 13 extinction sessions during which lever presses were not associated with alcohol deliveries and alcohol cues. During the test session, active lever presses were paired with alcohol deliveries, and lever presses were examined. We found that overexpression of BDNF in vlOFC to DLS neurons reduced total active lever presses (Fig. 4l), cumulative lever presses (Fig. 4m) and port entries (Fig. 4n) following a period of extinction (Average active lever presses: Mann–Whitney Test: U = 2, *p* = 0.0317; Cumulative presses: Two-way RM ANOVA: effect of virus F_(1,8)_ = 10.42, *p* = 0.0121; effect of session F_(1.642,13.14)_ = 17.13, *p* = 0.0004; effect of interaction F_(11,88)_ = 5.006, *p* < 0.001; Port entries: Mann–Whitney Test: U = 2, *p* = 0.0317). These behavioral changes were not due to an alteration in locomotion, as shown in Supplementary Fig. 6f (Mann–Whitney Test: U = 20, *p* = 0.396). Together, these data suggest that BDNF in vlOFC to DLS projecting neurons plays a role in alcohol seeking and relapse.

### Systemic administration of a TrkB agonist reverses habitual alcohol seeking

**Fig. 5:**
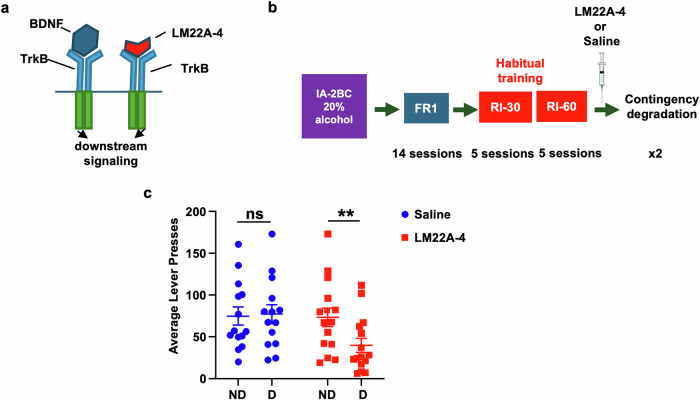
Systemic administration of TrkB agonist LM22A-4 biases habitually trained mice towards goal-directed alcohol seeking. **a** LM22A-4 mechanism of action: LM22A-4 binds to and activates TrkB, leading to the activation of downstream signaling pathways [65]. **b** Timeline of operant self-administration training. Following 7 weeks of IA2BC-20% alcohol in the home cage (Supplementary Table 1), mice began operant training for alcohol (20%) self-administration. Mice were trained on FR1 schedule followed by an initial habitual alcohol seeking training using random interval (RI) training schedule followed by contingency degradation testing. Mice were pseudo-randomly assigned and received an i.p. administration of saline or LM22A-4 (100 mg/kg) 30 min prior to the first contingency degradation. **c** Average lever presses of saline and LM22A-4 treated mice during ND and D testing sessions in RI schedule of reinforcement. Data are represented as mean ± SEM; **p < 0.01, ns: non-significant,  *n* = 14.

Finally, to examine the translational utility of the findings, and to determine if BDNF sigmaling alters alcohol habits, we utilized the BDNF receptor TrkB agonist, LM22A-4 which selectively binds and activates TrkB [65–68] (Fig. 5a) and tested its ability to suppress habitual alcohol seeking. A new cohort of mice underwent 7 weeks of IA20%2BC. Following the 7 weeks of IA20%2BC, mice were trained to self-administer 20% alcohol in operant chambers. After 7 initial training sessions on an FR1 schedule, mice began operant training using an RI schedule [53–55, 57] (Timeline, Fig. 5b, Supplementary Table 1). Mice were subjected to 5 sessions of RI30 and RI60 training followed by contingency degradation as described above (Timeline, Fig. 5b). Mice received an intraperitoneal (i.p.) administration of saline or a TrkB agonist, LM22A-4 (100 mg/kg) [29], 30 min prior to the degradation session, in a counterbalanced manner. As shown in Fig. 5c, RI-trained mice treated with saline showed no significant differences in lever pressing between non-degraded and degraded sessions. However, RI-trained mice treated with LM22A-4 exhibited a reduction in lever presses during contingency degradation, compared with non-degraded sessions (Fig. 5c) (Two-Way RM ANOVA, main effect of degradation F (1, 26) = 13.52, *p* = 0.0011, effect of treatment F(1,28) = 0.93, *p* = 0.342, effect of interaction F(1,26) = 16.08, *p* = 0.0005). Taken together, these data show that treatment with a TrkB agonist biases mice trained to habitually self-administer alcohol toward a goal-directed action selection strategy, suggesting that BDNF plays a role in moderating habitual alcohol. Furthermore, these results indicate the potential of utilizing a TrkB agonist to prevent habitual drug seeking.

## Discussion

Here, we investigated the potential role of BDNF in vlOFC-to-DLS circuit in alcohol-drinking and seeking behaviors. We found that chronic high alcohol intake decreases *BDNF* levels in the vlOFC but not in mOFC or M2 of male but not female mice. We further discovered that BDNF in a small ensemble of vlOFC neurons projecting to the DLS gates alcohol intake, seeking, and relapse. Finally, we showed that systemic administration of a TrkB agonist biases habitual alcohol seeking to goal-directed behavior, suggesting the utility of TrkB agonist to dampen habitual alcohol seeking.

### Long-term high levels of alcohol intake decrease BDNF levels in the vlOFC of males but not female mice

The rodent OFC is a complex brain region containing several functionally distinct subregions, with different neuronal populations extending axonal projections throughout the brain [35]. We recently showed that the vlOFC sends projections to the DLS [23]. In rodents, primates, and humans, the vlOFC is an anatomically separate subregion, with distinct corticostriatal circuits linked to specific behavioral functions [35, 69]. We found a significant decrease in *BDNF* mRNA levels in the vlOFC after high alcohol drinking in male mice. The mechanism responsible for the attenuation of *BDNF* levels in the vlOFC following long-term binge drinking and withdrawal remains unknown. *BDNF* mRNA is regulated in part by the microRNA (miR) machinery which promotes mRNA degradation or translation repression [70]. We, and others, previously showed that repeated cycles of high alcohol intake or alcohol vapor exposure and withdrawal decrease *BDNF* expression in the medial PFC (mPFC) of mice and rats [25, 26] which correlates with increased levels of miR-30a-5p and miR-206 both targeting *BDNF* mRNA [25, 26]. Overexpression of these miRs in the mPFC of rodents led to an escalation of alcohol intake while their inhibition reduced high drinking [25, 26]. These studies were conducted on male mice; however, evidence suggests sex-specific patterns of miR expression in response to various stimuli [71, 72]. Therefore, it would be of interest to identify the BDNF-targeting miRs increased in the vlOFC after high alcohol intake in male vs. female mice, which could be one of the mechanisms underlying the sexual dimorphism we identified in this study.

### BDNF in vlOFC to DLS circuitry controls alcohol drinking behaviors

We found that BDNF overexpression in vlOFC-to-DLS projecting neurons decreases alcohol drinking, seeking, and relapse. The OFC is implicated in motivation [35, 53] and modifying stimulus-outcome associations [35, 53, 56, 73, 74], along with reward seeking [35, 53]. Gourley and colleagues reported that BDNF in the vlOFC is involved in goal-directed decision-making in mice [56, 75]. Specifically, the authors showed that the knockdown of BDNF in OFC interferes with stimulus-outcome and response-outcome associations and that systemic administration of BDNF agonists rescues action-selection associations [76]. In addition, Pitts and colleagues found BDNF to be involved in the balance between action and habit [77] by showing that overexpressing truncated inactive TrkB in vlOFC impedes goal-directed action [77]. These findings, together with ours, suggest that BDNF in the vlOFC-to-DLS circuit may bias mice towards goal-directed alcohol seeking and that BDNF in vlOFC-to-DLS circuit may gate alcohol intake by altering decision-making. However, further work is required to address this question.

Axonal release of BDNF activates TrkB receptors in the target region. We previously reported that BDNF-positive vlOFC neurons form synapses with DLS neurons in mice [23], suggesting that TrkB signaling in the DLS is activated by overexpression of BDNF in vlOFC-to-DLS neurons. TrkB activation promotes the activation of ERK1/2, PLC/PKC, or PI3K/AKT signaling [6, 78]. We previously found that BDNF-mediated activation of TrkB in the DLS gates alcohol intake in a mechanism that depends on ERK1/2 but not PI3K/AKT or PLC/PKC signaling [19], suggesting that BDNF from vlOFC neurons promotes the activation of TrkB/ERK1/2 leading to transcriptional and/or translational modifications that in turn gate alcohol drinking behaviors. This line of research will be explored in future studies.

The majority of DLS neurons are dopamine D1 receptors (D1) or dopamine D2 receptors (D2)-expressing medium spiny neurons (MSN) [79]. Both D1 and D2 MSN express TrkB receptors [80]. Within the DLS, it is plausible that BDNF signaling via TrkB is differentially regulated depending on the target cell i.e. D1 or D2 MSNs [80–82]. Work from Nestler and colleagues suggests that activation of BDNF-TrkB signaling in D1 or D2 MSN in the nucleus accumbens generates opposite effects on cocaine and morphine-dependent rewarding behaviors in mice [80, 81]. Further studies are required to gain insight into BDNF-TrkB signaling in behavioral responses to alcohol in subpopulations of DLS neurons.

The vlOFC extends projections to several other brain regions, including the hippocampus, substantia nigra, ventral tegmental area, and several cortical subregions [83, 84]. In addition, work from Gourley and colleagues suggest that the vlOFC projections to the ventrolateral striatum, which includes the nucleus accumbens, regulate goal-directed food and cocaine seeking [56] and Saunders and colleagues recently reported a role of the vlOFC to basolateral amygdala circuit is critical during reward seeking [85]. Thus, we cannot exclude the possibility that BDNF originating from the vlOFC activates TrkB signaling in one or more of these brain regions and may also play a role in alcohol-mediated behaviors.

#### Translational implications

We previously reported that administration of LM22A-4 converts compulsive alcohol drinking of Met68BDNF mutant mice to moderate levels of intake [29]. We show herein that LM22A-4 reverses habitual alcohol seeking. Although it is likely that the reduction in habitual alcohol behavior by LM22A-4 is due to its stimulatory actions in the DLS, we cannot exclude the possibility that the attenuation of habit is mediated by TrkB in brain regions other than the DLS. LM22A-4 has been shown to have promising effects in preclinical studies for the treatment of several neurological diseases [86–89]. Thus, our data give rise to the potential use of LM22A-4 in AUD.

Together, our data highlight the importance of a small neuronal ensemble in regulating high alcohol intake, seeking, and relapse. Our findings also provide evidence for a potentially new drug target to combat AUD phenotypes, including habit.

## Supplementary information

Supplemental material and figures

## Data Availability

Any data not contained in the paper are available on request with appropriate agreements. Source data are provided with this paper.
