# Supplementary material for: BDNF in ventrolateral orbitofrontal cortex to dorsolateral striatum circuit moderates alcohol consumption, seeking and relapse
Source: Neuropsychopharmacology. 2025 Nov 9;51(3):565–76. doi: 10.1038/s41386-025-02274-1 (PMC12824369; doi:10.1038/s41386-025-02274-1)

BDNF in Ventrolateral Orbitofrontal Cortex to Dorsolateral Striatum Circuit Moderates Alcohol Consumption, Seeking and Relapse

Sowmya Gunasekaran, Jeffrey J. Moffat, Joshua D. Epstein, Khanhky Phamluong, Yann Ehinger and Dorit Ron

Supplementary Material and Methods

Reagents

Ethyl alcohol (190 proof) was purchased from VWR (Radnor, PA), and sucrose was purchased from Fisher Scientific (Pittsburgh, PA). The Qiazol RNA isolation kit was purchased from Qiagen (Redwood City, CA). cDNA was synthesized using the iScript cDNA Synthesis Kit (BioRad). Powerup SYBR Green PCR Master mix (ThermoFisher) was used for quantitative real-time PCR. LM22A-4 (N,N,N-tris [2-hydroxyethyl]-1,3,5-benzene tricarboxamide) was a generous gift from Drs. Masa (University of California San Francisco) and Lango (Standford University) who developed and synthesized LM22A-4 [1]. Other common reagents were from Sigma Aldrich (St. Louis, MO) or Fisher Scientific (Pittsburgh, PA).

Preparation of solutions

Alcohol solution was prepared from absolute anhydrous alcohol (190 proof) diluted to 20% alcohol (v/v) in tap water. Sucrose solution was diluted to 0.3% sucrose (v/v) in tap water. LM22A-4 solution was prepared in 0.9% saline.

Collection of brain samples for biochemical analyses

Mice were euthanized 4 hours and 24 hours after the last drinking session (binge and withdrawal time point). Brains were removed and dissected on an ice-cold platform into 1 mm sections, and specific subregions-vlOFC, mOFC, and motor cortex (M2) were dissected based on Allen Brain Atlas.

Quantitative real-time PCR

RNA was isolated using the RNeasy kit, and cDNA was synthesized using the iScript cDNA Synthesis Kit according to the manufacturer's instructions. The resulting cDNA was used for quantitative real-time PCR, using Powerup SYBR Green PCR Master mix. Thermal cycling was performed on QuantStudio 5 real-time PCR System (Thermo Fisher Scientific Inc.) using a relative calibration curve. The quantity of *BDNF* mRNA was measured and expressed relative to *GAPDH* mRNA. PCR primers used: *BDNF* Forward 5'- TGC AGG GGC ATA GAC AAA AGG-3', *BDNF* Reverse 5'- CTT ATG AAT CGC CAG CCA ATT CTC-3', *GAPDH* Forward 5'-CGA CTT CAA CAG CAA CTC CCA CTC TTC C-3' and *GAPDH* Reverse 5'- TGG GTG GTC CAG GGT TTC TTA CTC CTT-3'.

Adeno-associated viruses

AAV2-DIO-Ef1a-BDNF-IRES-mCherry virus (AAV2-DIO-BDNF-mCherry; $3x10^{12}$ vg/ml) and AAV2-DIO-Ef1a-mCherry virus (AAV2-DIO-mCherry; $1x10^{12}$ vg/ml) were constructed in conjunction with C&M Biolabs (Richmond, CA) and were produced by the Duke University Viral Vector Core. AAV2-retrograde(retro)-Cre-GFP virus (AAVretro.hSyn.HI.eGFP-Cre.WPRE.SV40; $3x10^{12}$ vg/ml) was purchased from Addgene.

Stereotaxic viral infection

Mice were anesthetized by vaporized isoflurane and placed on a digital stereotaxic frame (David Kopf Instruments). Two holes were drilled above the site of viral injection. The injectors (stainless tubing, 33 gauges; Small Parts Inc.) were slowly lowered into the target region. The injectors were connected to 10 µl Hamilton syringes, and the infusion was controlled by an automatic pump at a rate of 0.1 µl/min. The injectors remained in place for an additional 10 minutes to allow the virus to diffuse and then were slowly removed.

For circuit-specific expression of BDNF, mice received bilateral infusion of 1 µl of AAV2-DIO-Ef1a-BDNF-IRES-mCherry virus (AAV2-DIO-BDNF-mCherry, $3x10^{12}$ gc/ml) per hemisphere into the vlOFC (AP: +2.2, ML: ± 1.2, DV: -2.6) or M2 (AP: +2.2, ML: ± 1.3, DV: -1.35) and 1 µl of AAVretro-Cre-GFP ($3x10^{12}$ vg/ml) into the DLS (AP: +1.1, ML: ± 1.9, DV: -2.95) or DMS (AP +1.1, ML +/- 1.2, DV -2.95). Control animals received 1 µl of empty vector: AAV2-DIO-Ef1a-mCherry virus (AAV-DIO-mCherry ,$1x10^{12}$ gc/ml) in the vlOFC or M2 and 1 µl of AAVretro-Cre-GFP ($3x10^{12}$vg/ml) in the DLS or DMS.

Confirmation of viral expression

At the end of the experiments, animals were euthanized by cervical dislocation, the brains were removed, placed on ice, and dissected into 1 mm coronal sections. The fluorescent protein expressed by the virus (either GFP or mCherry) was visualized using an EVOS FL tabletop fluorescent microscope (ThermoFisher Scientific) or Fluoview FV31S-SW confocal microscope (Olympus) and images were obtained. Five animals that failed to exhibit fluorescence associated with viral overexpression were excluded from the study.

Behavioral procedures

Intermittent access to 0.3 % sucrose two-bottle choice

The sucrose intake procedure was conducted as previously described [2]. Animals received one bottle of 0.3% sucrose and one bottle of water 24 hours a day for two weeks on Monday, Wednesday, and Friday, with 24 or 48 hours (weekend) sucrose deprivation periods in which mice consumed only water. Sucrose solution intake (ml/kg), water intake (ml/kg), total fluid intake (ml/kg), and the preference ratio (volume of sucrose solution intake/total volume of fluid intake) were recorded every 24 hours for the duration of sucrose access. Corrections were made to account for spillage based on bottles affixed to an empty cage.

Open-field locomotion test

Open field locomotion test was conducted as described in [2]. Mice Specifically, mice were habituated to the room for 60 minutes prior to the experiment. Mice were placed in an open field apparatus (43cm x 43cm) in low-light conditions and allowed to explore for 10 minutes. Locomotor activity was tracked using EthoVision XT software (Noldus, Leesburg, VA), and total movement (cm) and velocity (cm/s) were recorded. At the end of the session, the mouse was removed, and the apparatus was cleaned between sessions.

Figure	Average Consumption (g/kg/24hr)	SEM (±g/kg/24hr)
1B-D and Sup. Fig 1A Males	14.47	1.00
1E-G and Sup. Fig 1D Females	19.27	1.10
2F AAV-DIO-mCherry	15.07	1.28
2F AAV-DIO-BDNF-mCherry	11.86	1.11
2I AAV-DIO-mCherry	91.62 (ml/kg)	9.62 (ml/kg)
2I AAV-DIO-BDNF-mCherry	101.27 (ml/kg)	15.69 (ml/kg)
3C AAV-DIO-mCherry	15.86	1.54
3C AAV-DIO-BDNF-mCherry	18.02	1.67
3G AAV-DIO-mCherry	11.81	0.86
3G AAV-DIO-BDNF-mCherry	14.79	1.87
5C-D TrkB agonist	16.51	0.79
Sup. Fig 7A AAV-DIO-mCherry	13.89	1.52
Sup. Fig 7A AAV-DIO-BDNF-mCherry	15.39	2.45

Supplementary Table S1. Average alcohol and sucrose consumption

Mice were subjected to 7 weeks of IA20%BC or 7 sessions of IA0.3%Sucrose. The average alcohol intake (g/kg) and sucrose intake (ml/kg) were calculated.

Supplementary Figures

Supplementary Figure 1. Drinking profile of male and female mice

Mice were subjected to IA20%2BC for 7 weeks in the home cage before harvesting vlOFC, mOFC and M2 regions for biochemical analysis. Alcohol consumption, preference, and water consumption of male (**a-c**) and female (**d-f**) mice.

Supplementary Figure 2. Confirmation of the Cre/DIO strategy efficacy

(**a**) Schematic representation of BDNF overexpression in vlOFC-to-DLS circuit. Mice received bilateral injections of AAVretro-Cre-GFP in the DLS. (**b**) Representative images of GFP expression in mice bilaterally infected with AAVretro-Cre-GFP in the DLS without infection of cortical regions with AAV-DIO-mCherry or AAV-DIO-BDNF-mCherry. Top left panel and bottom left panel depicts GFP (green) and DAPI (cyan). Top and bottom right panels depict mCherry (red) and GFP (green). (**c**) Mice received bilateral injections of AAV2-DIO-BDNF-mCherry in vlOFC without a parallel infection of the DLS or DMS with AAVretro-Cre-GFP. (**d**) Representative images showing no mCherry expression following AAV2-DIO-BDNF-mCherry infusion in the vlOFC without AAVretro-Cre-GFP infusion in the DLS. Top left panel and bottom left panel depict mCherry (red) and DAPI (cyan). Top and bottom right panels depict mCherry (red) and GFP (green).

Supplementary Figure 3. Confirmation of viral spread in vlOFC-DLS circuit

(**a**) Schematic representation of bilateral injections of AAV-DIO-BDNF-mCherry in vlOFC and AAVretro-Cre-GFP in the DLS. **(b)** AAV-DIO-BDNF-mCherry was infused in vlOFC and the distribution of mCherry labelled BDNF-positive neurons was examined in the rostrocaudal axis. The left panel depicts mCherry (red) and the right panel depicts GFP (green). **(c)** AAVretro-Cre-GFP was infused in the DLS and the distribution of the retrograde GFP labelled Cre expression was examined in rostrocaudal axis. The left panel depicts mCherry (red) and the right panel

depicts GFP (green). Scale bar is indicated on each panel. vlOFC: ventrolateral orbitofrontal cortex; DLS: dorsolateral striatum.

Supplementary Figure 4. Overexpression of BDNF in vlOFC-to-DLS projecting neurons does not alter water or total fluid consumption during alcohol and sucrose intake

(**a-b**) Water (**a**) and total fluid consumption **(b)** of alcohol was measured after BDNF overexpression in vlOFC-to-DLS projecting neurons. (**c-d**) Water **(c)** and total consumption **(d)** of sucrose was measured after BDNF overexpression in vlOFC-to-DLS projecting neurons. Data are represented as mean ± SEM, n=6-9 per group.

Supplementary Figure 5. Overexpression of BDNF in vlOFC-to-DLS projecting neurons does not alter locomotion

(**a**) Experimental timeline: Mice received bilateral injections of AAV2-DIO-BDNF-mCherry in the vlOFC and AAVretro-Cre-GFP in the DLS. Three weeks after the surgery, mice were subjected to the open field test. (**b**) Representative tracks of the movement of AAV2-DIO-mCherry and AAV2-DIO-BDNF-mCherry mice. (**c-d**) Locomotion was recorded for 10 minutes, and total distance traveled (**c**), and average velocity (**d**) were calculated. Data are represented as mean ± SEM. ns: non-significant. n=6-7 per group.

Supplementary Figure 6. Overexpression of BDNF in vlOFC-to-DMS or M2-to-DLS neurons does not alter water and total fluid consumption during voluntary alcohol intake in mice

(**a-b**)**.** Water consumption **(a)** and total fluid consumption **(b)** was calculated after BDNF overexpression in vlOFC-to-DMS circuit. (**c-d**) Water consumption (**c**) and total fluid consumption (**d**) was calculated after BDNF overexpression in M2-to-DLS circuit**.** Data are represented as mean ± SEM, n=8-10 per group.

Supplementary Figure 7. Drinking profile of mice prior to operant self-administration training, average lever presses of mice in operant training before undergoing surgery and open field test after surgery

Mice were subjected to IA20%2BC for 7 weeks in the home cage and were assigned to AAV-DIO-mCherry and AAV-DIO-BDNF-mCherry groups before they were trained to operantly self-administer alcohol. Alcohol consumption (**a**), preference (**b**), water consumption (**c**) and total fluid (**d**) were recorded. (**e**) The baseline averages of active lever presses during FR1, RI30 and RI60 sessions in operant self-administration training before viral infection. (**f**) Open field locomotion test. Mice were placed in an open field and total distance was recorded for 10 minutes. n=5 per group.

References

1 Massa, S. M. *et al.* Small molecule BDNF mimetics activate TrkB signaling and prevent neuronal degeneration in rodents. *J Clin Invest* **120**, 1774-1785, doi:41356 [pii] 10.1172/JCI41356 (2010).

2 Hoisington, Z. W. *et al.* The Small G-Protein Rac1 in the Dorsomedial Striatum Promotes Alcohol-Dependent Structural Plasticity and Goal-Directed Learning in Mice. *J Neurosci* **44**, doi:10.1523/JNEUROSCI.1644-23.2024 (2024).

Supplementary Figure 1. Drinking profiles of male and female mice

Males

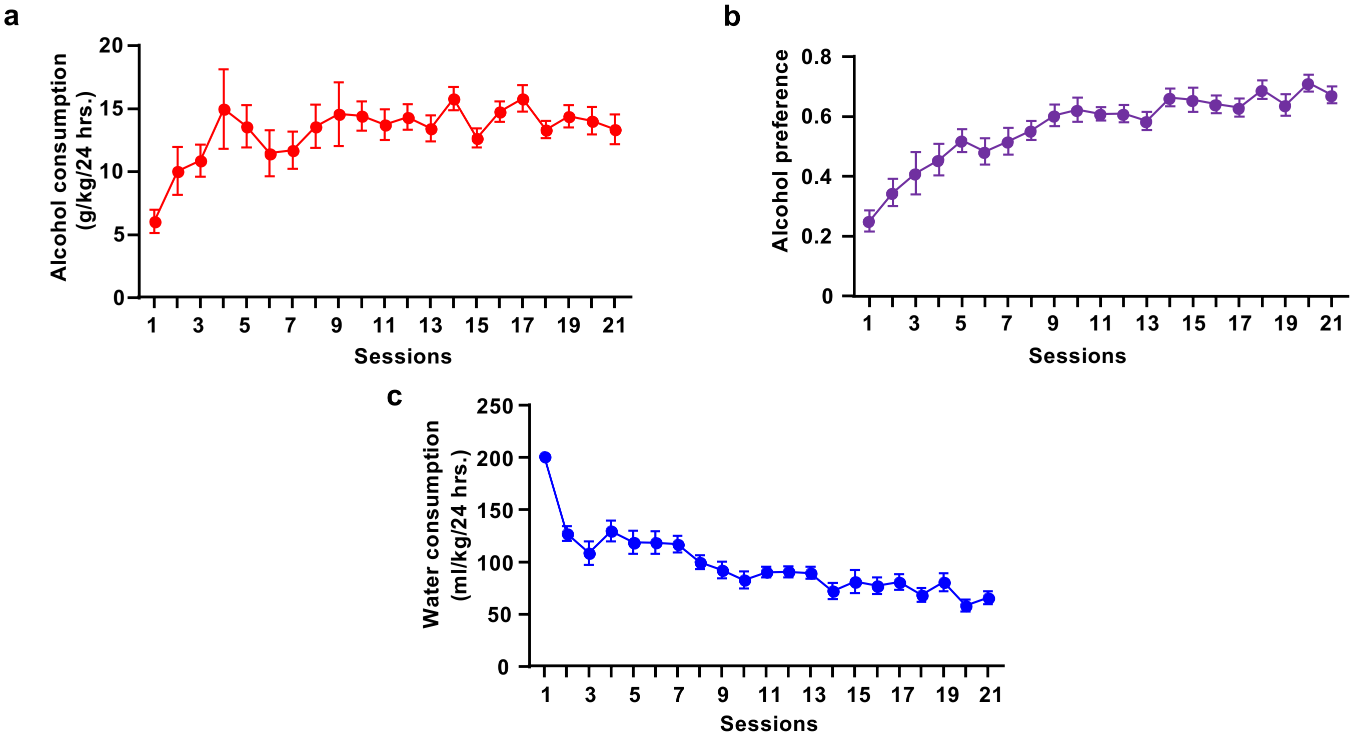

Females

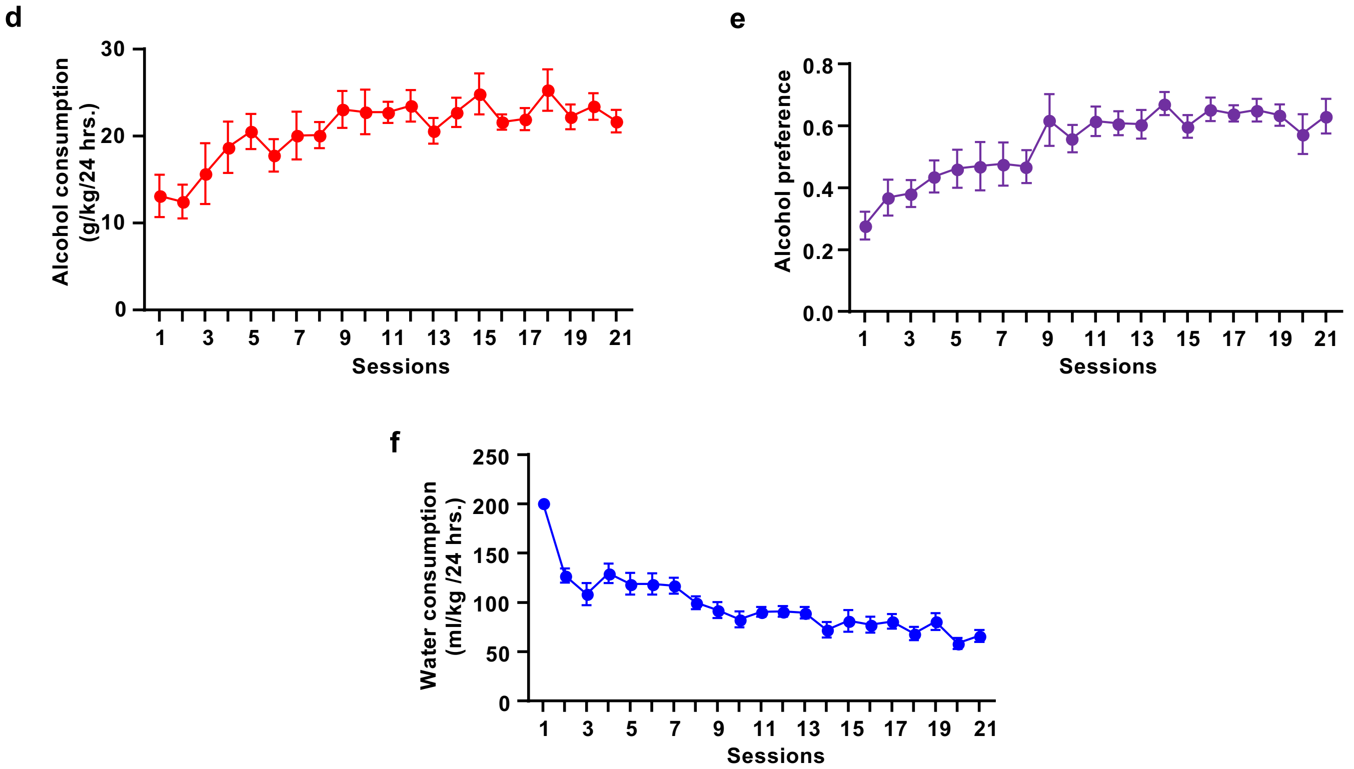

Supplementary Figure 2. Confirmation of the Cre/DIO strategy

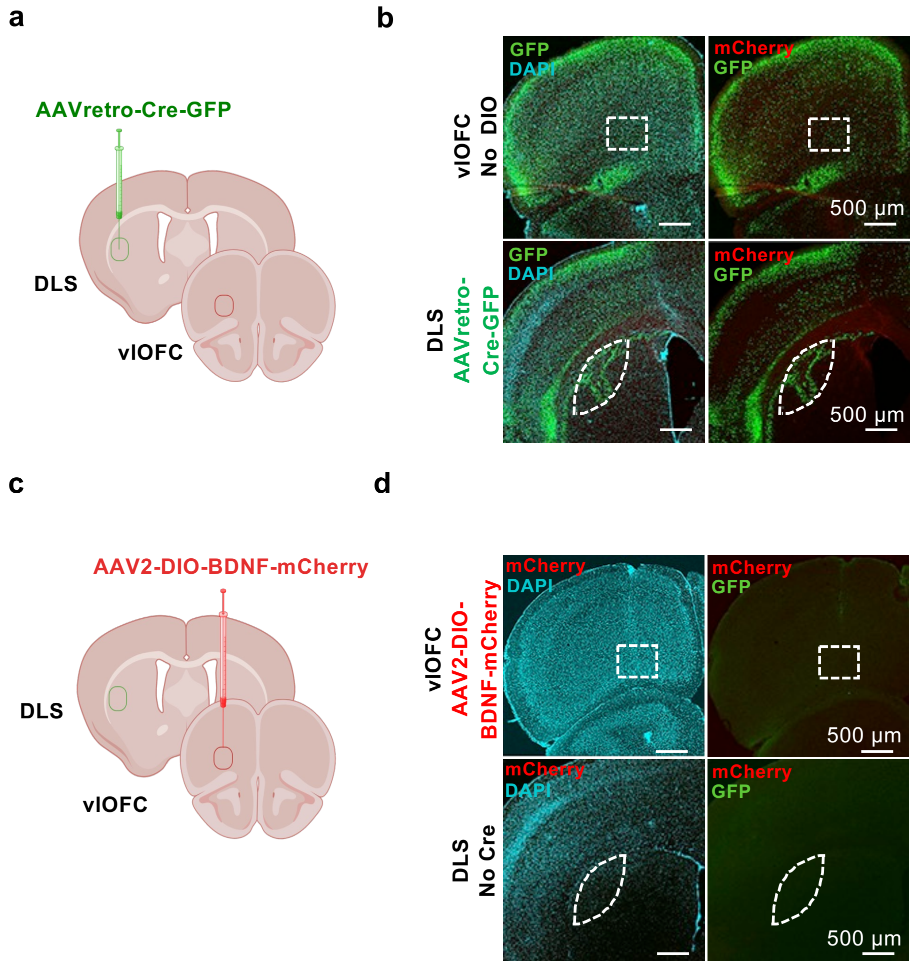

Supplementary Figure 3. Confirmation of viral spread in vlOFC-DLS circuit

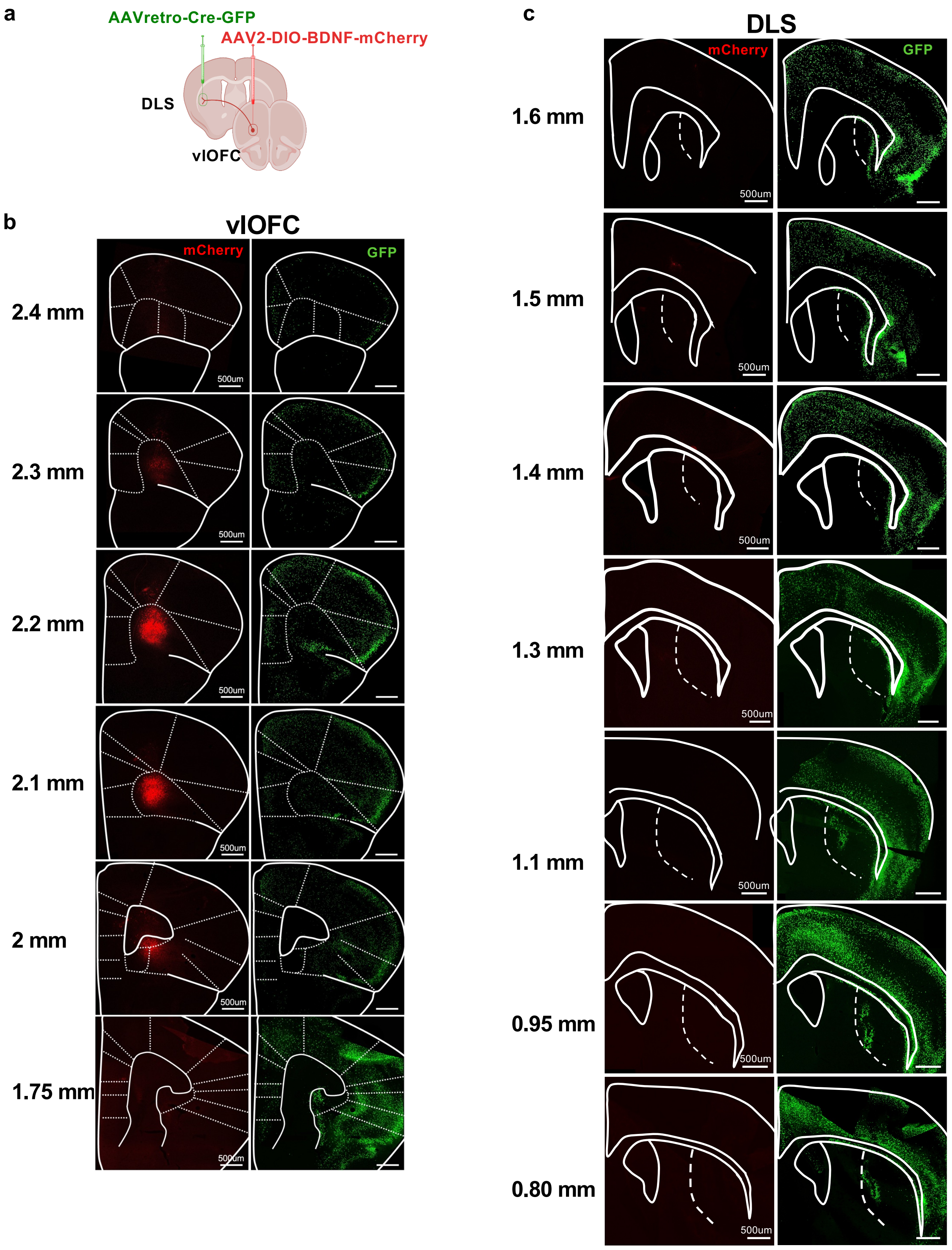

Supplementary Figure 4. Overexpression of BDNF in vlOFC to DLS projecting neurons does not alter water or total fluid consumption during alcohol and sucrose intake

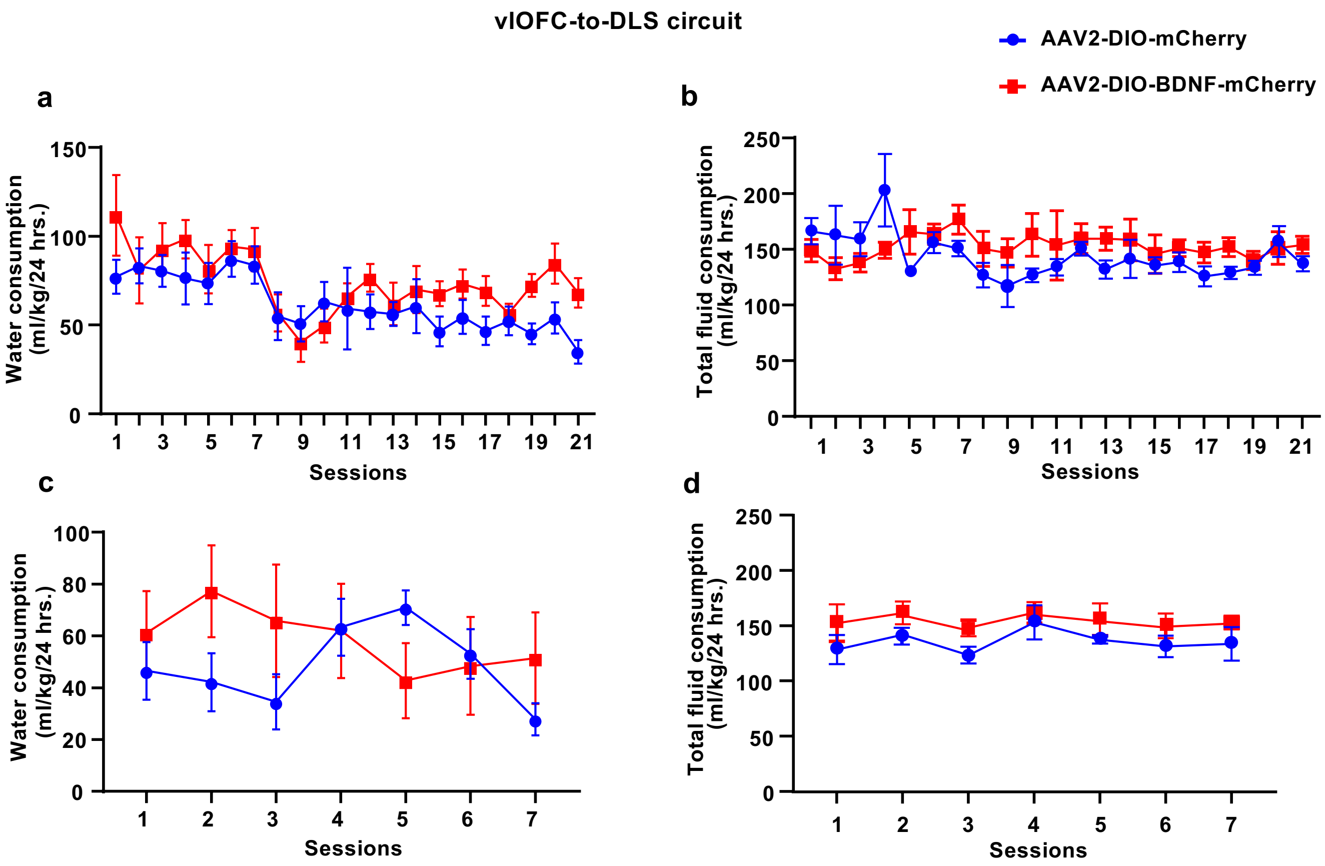

Supplementary Figure 5. Overexpression of BDNF in vlOFC to DLS projecting neurons does not alter locomotion

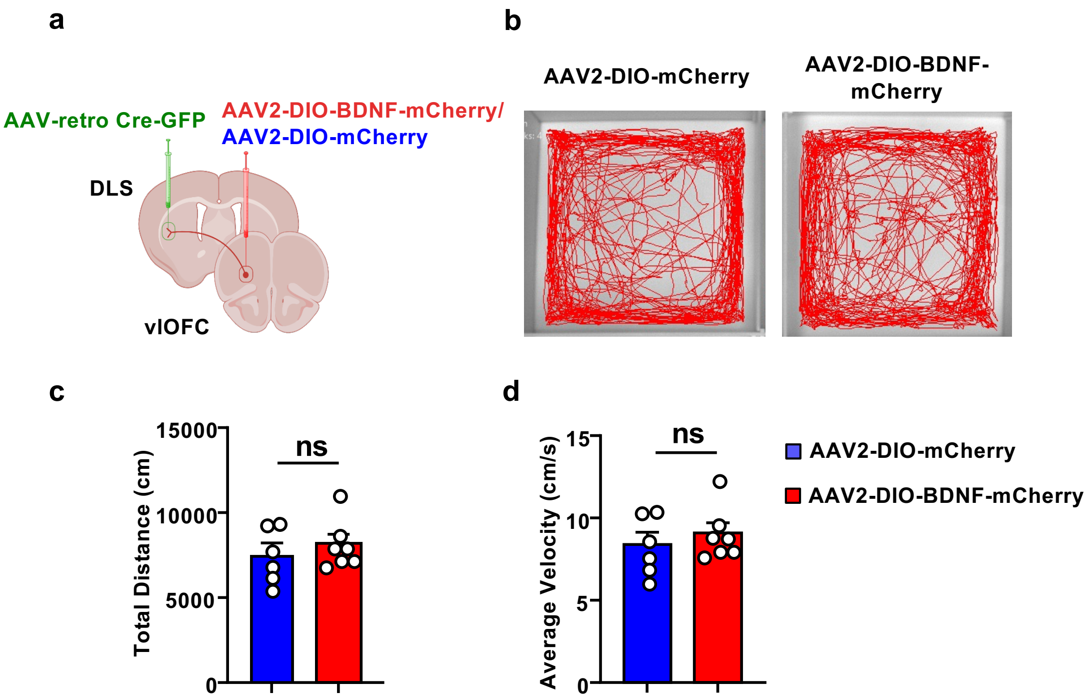

Supplementary Figure 6. Overexpression of BDNF in vlOFC to DMS or M2 to DLS neurons does not alter water and total fluid consumption during voluntary alcohol intake in mice

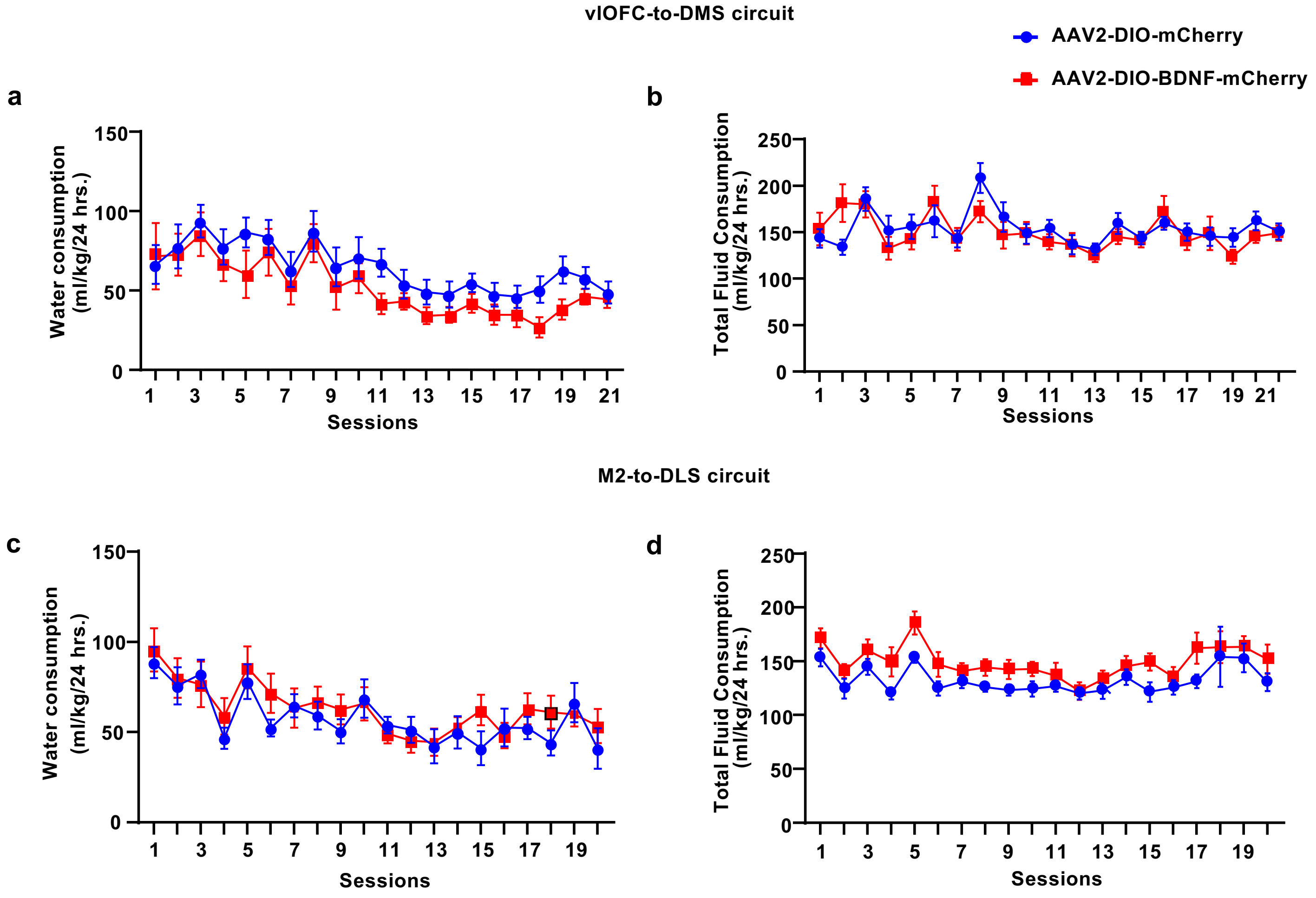

Supplementary Figure 7. Drinking profile of mice prior to operant self-administration training ,average lever presses of mice in operant training before undergoing surgery and open field test after surgery

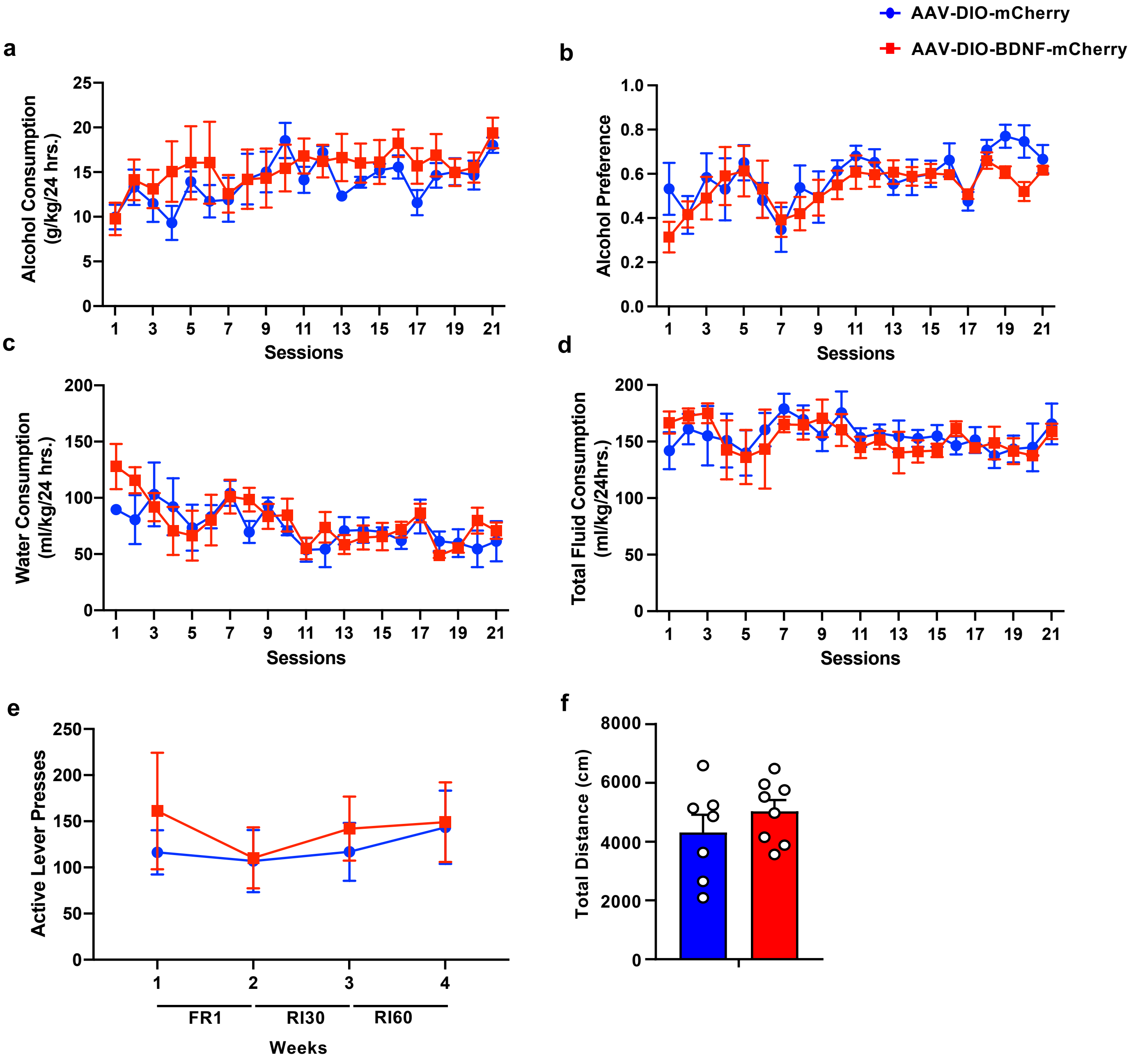

Supplement: Supplementary file 1 — Supplemental material and figures [file 41386_2025_2274_MOESM1_ESM.pdf]
